# Supplementary material for: Discovery and application of insertion-deletion (INDEL) polymorphisms for QTL mapping of early life-history traits in Atlantic salmon
Source: BMC Genomics. 2010 Mar 8;11:156. doi: 10.1186/1471-2164-11-156 (PMC2838853; doi:10.1186/1471-2164-11-156)
Supplement: Additional file 2 — Information on developed 76 locus single-run INDEL panel in Atlantic salmon. Information on fluorescence labeling, primer concentrations, PCR pooling and links to alignments, INDEL motifs and GENESCAN (Burge and Karlin 1997) predictions of genes/exons are available in html format. [file 1471-2164-11-156-S2.ZIP › Additionalfile2/table20742.html]

Contig Alignment

```
Base	A B C D   
1	. A . .    
2	. T . .    
3	. A . .    
4	. A . .    
5	. A . .    
6	. T . .    
7	. G . .    
8	. T . .    
9	. A . .    
10	. A . .    
11	. T . .    
12	. A . .    
13	. A . .    
14	. G . .    
15	. A . .    
16	. T . .    
17	. T . .    
18	. T . .    
19	. A . .    
20	. T . .    
21	. T . .    
22	. A . .    
23	. A . .    
24	. G . .    
25	. T . .    
26	. A . .    
27	. T . .    
28	. C . .    
29	. T . .    
30	. T . .    
31	. A . .    
32	. A . .    
33	. G . .    
34	. C . .    
35	. A . .    
36	. T . .    
37	. T . .    
38	. T . .    
39	. T . .    
40	. C . .    
41	. C . .    
42	. A . .    
43	. C . .    
44	. T . .    
45	. T . .    
46	A A . .    
47	T T . .    
48	G G . .    
49	C C . .    
50	A A . .    
51	T T . .    
52	T T . .    
53	T T . .    
54	A A . .    
55	A A . .    
56	A A . .    
57	T T . .    
58	G G . .    
59	A A . .    
60	T T . .    
61	T T . .    
62	T T . .    
63	C C . .    
64	T T . .    
65	C C . .    
66	T T . .    
67	T T . .    
68	T T . .    
69	T T . .    
70	T T . .    
71	A A . .    
72	C C . .    
73	A A . .    
74	T T . .    
75	A A . .    
76	A A . .    
77	T T . .    
78	G G . .    
79	G G . .    
80	A A . .    
81	C C . .    
82	A A . .    
83	A A . .    
84	T T . .    
85	T T . .    
86	A A . .    
87	G G . .    
88	A A . .    
89	T T . .    
90	C C . .    
91	T T . .    
92	A A . .    
93	A A . .    
94	A A . .    
95	C C . .    
96	G G . .    
97	T T . .    
98	A A . .    
99	G G . .    
100	C C . .    
101	C C . .    
102	C C . .    
103	A A . .    
104	A A . .    
105	A A . .    
106	A A . .    
107	T T . .    
108	G G . .    
109	T T . .    
110	A A . .    
111	C C . .    
112	A A . .    
113	G G . .    
114	G G . .    
115	C C . .    
116	A A . .    
117	A A . .    
118	T T . .    
119	A A . .    
120	T T . .    
121	A A . .    
122	G G . .    
123	A A . .    
124	A A . .    
125	C C . .    
126	T T . .    
127	T T . .    
128	C C . .    
129	C C . .    
130	A A . .    
131	C C . .    
132	T T . .    
133	T T . .    
134	T T . .    
135	C C . .    
136	T T . .    
137	C C . .    
138	C C . .    
139	A A . .    
140	T T . .    
141	A A . .    
142	G G . .    
143	G G . .    
144	G G . .    
145	A A . .    
146	A A . .    
147	A A . .    
148	A A . .    
149	C C . .    
150	A A . .    
151	A A . .    
152	A A . .    
153	C C . .    
154	G G . .    
155	T T . .    
156	T T . .    
157	G G . .    
158	T T . .    
159	G G . .    
160	G G . .    
161	A A . .    
162	A A . .    
163	A A . .    
164	G G . .    
165	A A . .    
166	A A . .    
167	C C . .    
168	T T . .    
169	C C . .    
170	T T . .    
171	G G . .    
172	A A . .    
173	A A . .    
174	A A . .    
175	G G . .    
176	C C . .    
177	T T T .    
178	C C C .    
179	T T T .    
180	C C C .    
181	C C C .    
182	C C C .    
183	G G G .    
184	C C C .    
185	G G G .    
186	G G G .    
187	T T T .    
188	A A A .    
189	T T T .    
190	A A A .    
191	C C C .    
192	A A A .    
193	G G G .    
194	T T T .    
195	A A A .    
196	C C C .    
197	T T T .    
198	G G G .    
199	T T T .    
200	A A A .    
201	C C C .    
202	A A A .    
203	T T T .    
204	G G G .    
205	G G G .    
206	T T T .    
207	C C C .    
208	A A A .    
209	C C C .    
210	A A A .    
211	G G G .    
212	A A A .    
213	A A A .    
214	A A A .    
215	A A A .    
216	C C C .    
217	A A A .    
218	G G G .    
219	C C C .    
220	T T T .    
221	G G G .    
222	G G G .    
223	C C C .    
224	A A A .    
225	T T T .    
226	A A A .    
227	G G G .    
228	A A A .    
229	A A A .    
230	A A A .    
231	T T T .    
232	C C C .    
233	T T T .    
234	A A A .    
235	C C C .    
236	A A A .    
237	T T T .    
238	A A A .    
239	T T T .    
240	G G G .    
241	A A A .    
242	A A A .    
243	C C C .    
244	C C C .    
245	A A A .    
246	A A A .    
247	A A A .    
248	A A A .    
249	T T T .    
250	C C C .    
251	A A A .    
252	C C C .    
253	C C C .    
254	A A A .    
255	A A A .    
256	T T T .    
257	C C C .    
258	T T T .    
259	A A A .    
260	C C C .    
261	A A A .    
262	T T T .    
263	G G G .    
264	C C C .    
265	A A A .    
266	A A A .    
267	A A A .    
268	A A A .    
269	A A A .    
270	C C C .    
271	A A A .    
272	G G G .    
273	G G G .    
274	C C C .    
275	A A A .    
276	A A A .    
277	A A A .    
278	T T T .    
279	C C C .    
280	T T T .    
281	T T T .    
282	T T T .    
283	A A A .    
284	A A A .    
285	A A A .    
286	A A A .    
287	T T T .    
288	A A A .    
289	C C C .    
290	A A A .    
291	C C C .    
292	T T T .    
293	A A A .    
294	A A A .    
295	A A A .    
296	T T T .    
297	C C C .    
298	C C C .    
299	A A A .    
300	T T T .    
301	T T T .    
302	A A A .    
303	T T T .    
304	T T T .    
305	G G G .    
306	A A A .    
307	A A A .    
308	T T T .    
309	G G G .    
310	T T T .    
311	G G G .    
312	T T T .    
313	A A A .    
314	C C C .    
315	A A A .    
316	T - - .  1	 indel  
317	A - - .  1	 indel  
318	C - - .  1	 indel  
319	A - - .  1	 indel  
320	T T T .    
321	A A A .    
322	C C C .    
323	T T T .    
324	G G G .    
325	T T T .    
326	T T T .    
327	C C C .    
328	C C C .    
329	A A A .    
330	T T T .    
331	T T T .    
332	C C C .    
333	C C C .    
334	A A A .    
335	C C C .    
336	A A A .    
337	C C C .    
338	T T T .    
339	T T T .    
340	T T T .    
341	T T T .    
342	T T T .    
343	A A A .    
344	A A A .    
345	A A A .    
346	A A A .    
347	A A A .    
348	A A A .    
349	T T T .    
350	G G G .    
351	T T T .    
352	T T T .    
353	G G G .    
354	T T T .    
355	G G G .    
356	A A A .    
357	C C C .    
358	T T T .    
359	A A A .    
360	C C C .    
361	C C C .    
362	A A A .    
363	T T T .    
364	G G G .    
365	T T T .    
366	C C C .    
367	C C C .    
368	A A A .    
369	T T T .    
370	T T T .    
371	C C C .    
372	T T T .    
373	T T T .    
374	C C C .    
375	C C C .    
376	T T T .    
377	C C C .    
378	C C C .    
379	C C C .    
380	A A A .    
381	A A A .    
382	A A A .    
383	G G G .    
384	A A A .    
385	C C C .    
386	A A A .    
387	G G G .    
388	A A A .    
389	C C C .    
390	A G G .  1	 transition  
391	C C C .    
392	A A A .    
393	C C C .    
394	A A A .    
395	C C C .    
396	A A A .    
397	T T T .    
398	A A A .    
399	A A A .    
400	G G G .    
401	A A A .    
402	A A A .    
403	C C C .    
404	A A A .    
405	T T T .    
406	T T T .    
407	C C C .    
408	A A A .    
409	T T T .    
410	T T T .    
411	C C C .    
412	A A A .    
413	G G G .    
414	A A A .    
415	T T T .    
416	T T T .    
417	A A A .    
418	A A A .    
419	G G G .    
420	T T T .    
421	G G G .    
422	A A A .    
423	A A A .    
424	G G G .    
425	A A A .    
426	G G G .    
427	G G G .    
428	G G G .    
429	A A A .    
430	C C C .    
431	A A A .    
432	C C C .    
433	A A A .    
434	G G G .    
435	T T T .    
436	C C C .    
437	A A A .    
438	G G G .    
439	G G G .    
440	T T T .    
441	G G G .    
442	C C C .    
443	T T T .    
444	G G G .    
445	T T T .    
446	T T T .    
447	T T T .    
448	T T T .    
449	T T T .    
450	G G G .    
451	C C C .    
452	G G G .    
453	A A A .    
454	G G G .    
455	T T T .    
456	G G G .    
457	C C C .    
458	T T T .    
459	A A A .    
460	T T T .    
461	G G G .    
462	T T T .    
463	A A A .    
464	C C C .    
465	A A A .    
466	G G G .    
467	T T T .    
468	T T T .    
469	G G G .    
470	T T T .    
471	A A A .    
472	A A A .    
473	A A A .    
474	C C C .    
475	C C C .    
476	A A A .    
477	G G G .    
478	T T T .    
479	T T T .    
480	T T T .    
481	T T T .    
482	G G G .    
483	C C C .    
484	T T T .    
485	T T T .    
486	T T T .    
487	A A A .    
488	A A A .    
489	T T T .    
490	T T T .    
491	G G G .    
492	G G G .    
493	G G G .    
494	G G G .    
495	T T T .    
496	T T T .    
497	A A A .    
498	T T T .    
499	T T T .    
500	A A A .    
501	A A A .    
502	G G G .    
503	T T T .    
504	A A A .    
505	G G G .    
506	C C C .    
507	A A A .    
508	T T T .    
509	A A A .    
510	T T T .    
511	C C C .    
512	A A A .    
513	A A A .    
514	A A A .    
515	G G G .    
516	T T T .    
517	G G G .    
518	C C C .    
519	T T T .    
520	A A A .    
521	G G G .    
522	A A A .    
523	A A A .    
524	G G G .    
525	T T T .    
526	T T T .    
527	A A A .    
528	A A A .    
529	A A A .    
530	A A A .    
531	G G G .    
532	T T T .    
533	T T T .    
534	G G G .    
535	A A A .    
536	A A A .    
537	T T T .    
538	C C C .    
539	T T T .    
540	C C C .    
541	A A A .    
542	G G G .    
543	C C C .    
544	T T T .    
545	A A A .    
546	C C C .    
547	A A A .    
548	C C C .    
549	A A A .    
550	G G G .    
551	G G G .    
552	T T T .    
553	C C C .    
554	G G G .    
555	T T T .    
556	G G G .    
557	T T T .    
558	A A A .    
559	A A A .    
560	T T T .    
561	A A A .    
562	G G G .    
563	T T T .    
564	G G G .    
565	C C C .    
566	A A A .    
567	G G G .    
568	T T T .    
569	G G G .    
570	G G G .    
571	A A A .    
572	C C C .    
573	G G G .    
574	G G G .    
575	C C C .    
576	T T T .    
577	A A A .    
578	A A A .    
579	C C C .    
580	A A A .    
581	T T T .    
582	G G G .    
583	A A A .    
584	G G G .    
585	A A A .    
586	A A A .    
587	T T T .    
588	C C C .    
589	T T T .    
590	A A A .    
591	G G G .    
592	A A A .    
593	T T T .    
594	A A A .    
595	G G G .    
596	A A A .    
597	G G G .    
598	T T T .    
599	T T T .    
600	G G G .    
601	A A A .    
602	T T T .    
603	A A A .    
604	T T T .    
605	A A A .    
606	C C C .    
607	C C C .    
608	A A A .    
609	G G G .    
610	A A A .    
611	T T T .    
612	A A A .    
613	A A A .    
614	T T T .    
615	T T T .    
616	C C C .    
617	A A A .    
618	T T T .    
619	A A A .    
620	T T T .    
621	G G G .    
622	G G G .    
623	G G G .    
624	T T T .    
625	G G G .    
626	G G G .    
627	T T T .    
628	G G G .    
629	G G G .    
630	A A A .    
631	A A A .    
632	A A A .    
633	. A A .    
634	. A A .    
635	. A A .    
636	. A A .    
637	. A A .    
638	. G G .    
639	. A A .    
640	. G G .    
641	. G G .    
642	. A A .    
643	. T T .    
644	. A A .    
645	. A A .    
646	. A A .    
647	. A A .    
648	. T T .    
649	. G G .    
650	. G G .    
651	. C C .    
652	. G G .    
653	. T T .    
654	. G G .    
655	. A A .    
656	. C C .    
657	. T T .    
658	. G G .    
659	. C C .    
660	. C C .    
661	. C C .    
662	. G G .    
663	. A A .    
664	. C C .    
665	. C C .    
666	. A A .    
667	. T T .    
668	. G G .    
669	. C C .    
670	. C C .    
671	. G G .    
672	. T T .    
673	. T T .    
674	. T T .    
675	. A A .    
676	. A A .    
677	. C C .    
678	. C C .    
679	. C C .    
680	. C C .    
681	. A A .    
682	. A A .    
683	. A A .    
684	. C C .    
685	. T T .    
686	. G G .    
687	. C C .    
688	. C C .    
689	. A A .    
690	. C C .    
691	. A A .    
692	. T T .    
693	. T T .    
694	. C C .    
695	. A A .    
696	. C C .    
697	. T T .    
698	. T T .    
699	. C C .    
700	. A A .    
701	. A A .    
702	. T T .    
703	. G G .    
704	. C C .    
705	. T T .    
706	. C C .    
707	. T T .    
708	. A A .    
709	. C C .    
710	. C C .    
711	. A A .    
712	. T T .    
713	. G G .    
714	. A A .    
715	. C C .    
716	. A A .    
717	. C C .    
718	. T T .    
719	. G G .    
720	. C C .    
721	. C C .    
722	. G G .    
723	. A A .    
724	. A A .    
725	. G G .    
726	. G G .    
727	. C C .    
728	. C C .    
729	. T T .    
730	. T T .    
731	. A A .    
732	. G G .    
733	. . T .    
734	. . T .    
735	. . A .    
736	. . A .    
737	. . C .    
738	. . A A    
739	. . A A    
740	. . G G    
741	. . A A    
742	. . T T    
743	. . C C    
744	. . A A    
745	. . C C    
746	. . T T    
747	. . G G    
748	. . C C    
749	. . T T    
750	. . G G    
751	. . C C    
752	. . A A    
753	. . A A    
754	. . C C    
755	. . A A    
756	. . C C    
757	. . A A    
758	. . A A    
759	. . A A    
760	. . C C    
761	. . A A    
762	. . A A    
763	. . G G    
764	. . A A    
765	. . T T    
766	. . A A    
767	. . T T    
768	. . G G    
769	. . C C    
770	. . C C    
771	. . T T    
772	. . C C    
773	. . A A    
774	. . C C    
775	. . T T    
776	. . A A    
777	. . T T    
778	. . A A    
779	. . T T    
780	. . A A    
781	. . G G    
782	. . C C    
783	. . T T    
784	. . A A    
785	. . C C    
786	. . T T    
787	. . T T    
788	. . T T    
789	. . A A    
790	. . T T    
791	. . T T    
792	. . A A    
793	. . C C    
794	. . A A    
795	. . C C    
796	. . A A    
797	. . T T    
798	. . A A    
799	. . C C    
800	. . A A    
801	. . T T    
802	. . A A    
803	. . G G    
804	. . A A    
805	. . T T    
806	. . A A    
807	. . C C    
808	. . T T    
809	. . C C    
810	. . C C    
811	. . A A    
812	. . G G    
813	. . G G    
814	. . A A    
815	. . G G    
816	. . C C    
817	. . A A    
818	. . A A    
819	. . T T    
820	. . T T    
821	. . T T    
822	. . A A    
823	. . C C    
824	. . A A    
825	. . G G    
826	. . T T    
827	. . G G    
828	. . G A  1	 transition  
829	. . T T    
830	. . T T    
831	. . G G    
832	. . T T    
833	. . G G    
834	. . C C    
835	. . C C    
836	. . T T    
837	. . G G    
838	. . C C    
839	. . T T    
840	. . A A    
841	. . T T    
842	. . G G    
843	. . G G    
844	. . C C    
845	. . T T    
846	. . C C    
847	. . C C    
848	. . T T    
849	. . G G    
850	. . C C    
851	. . T T    
852	. . C C    
853	. . T T    
854	. . G G    
855	. . C C    
856	. . C C    
857	. . A A    
858	. . T T    
859	. . T T    
860	. . A A    
861	. . G G    
862	. . T T    
863	. . A A    
864	. . A A    
865	. . A A    
866	. . A A    
867	. . A A    
868	. . A A    
869	. . T T    
870	. . A A    
871	. . T T    
872	. . A A    
873	. . T T    
874	. . A A    
875	. . T T    
876	. . A A    
877	. . T T    
878	. . A A    
879	. . A A    
880	. . A A    
881	. . T T    
882	. . C C    
883	. . G G    
884	. . C C    
885	. . G G    
886	. . G G    
887	. . T T    
888	. . A A    
889	. . A A    
890	. . T T    
891	. . A A    
892	. . T T    
893	. . G G    
894	. . T T    
895	. . A A    
896	. . G G    
897	. . C C    
898	. . C C    
899	. . A A    
900	. . C C    
901	. . T T    
902	. . T T    
903	. . G G    
904	. . A A    
905	. . G G    
906	. . T T    
907	. . A A    
908	. . T T    
909	. . T T    
910	. . G G    
911	. . T T    
912	. . A A    
913	. . G G    
914	. . T T    
915	. . T T    
916	. . T T    
917	. . T T    
918	. . A A    
919	. . C C    
920	. . T T    
921	. . C C    
922	. . C C    
923	. . T T    
924	. . G G    
925	. . T T    
926	. . G G    
927	. . T T    
928	. . T T    
929	. . A A    
930	. . A A    
931	. . A A    
932	. . C C    
933	. . T T    
934	. . G G    
935	. . A A    
936	. . G G    
937	. . T T    
938	. . G G    
939	. . G G    
940	. . T T    
941	. . T T    
942	. . C C    
943	. . A A    
944	. . G G    
945	. . T T    
946	. . T T    
947	. . T T    
948	. . T T    
949	. . G G    
950	. . G G    
951	. . G G    
952	. . A A    
953	. . A A    
954	. . A A    
955	. . A A    
956	. . G G    
957	. . T T    
958	. . C C    
959	. . C C    
960	. . T T    
961	. . C C    
962	. . A A    
963	. . C C    
964	. . A A    
965	. . G G    
966	. . T T    
967	. . T T    
968	. . A A    
969	. . T T    
970	. . C A  1	 transversion  
971	. . . C    
972	. . . T    
973	. . . T    
974	. . . T    
975	. . . A    
976	. . . C    
977	. . . A    
978	. . . T    
979	. . . C    
980	. . . A    
981	. . . C    
982	. . . C    
983	. . . T    
984	. . . G    
985	. . . G    
986	. . . T    
987	. . . T    
988	. . . C    
989	. . . A    
990	. . . G    
991	. . . A    
992	. . . T    
993	. . . G    
994	. . . T    
995	. . . G    
996	. . . C    
997	. . . A    
998	. . . T    
999	. . . G    
1000	. . . G    
1001	. . . T    
1002	. . . G    
1003	. . . G    
1004	. . . A    
1005	. . . A    
1006	. . . C    
1007	. . . T    
1008	. . . G    
1009	. . . G    
1010	. . . T    
1011	. . . A    
1012	. . . A    
1013	. . . T    
1014	. . . G    
1015	. . . C    
1016	. . . C    
1017	. . . A    
1018	. . . A    
1019	. . . C    
1020	. . . A    
1021	. . . T    
1022	. . . T    
1023	. . . T    
1024	. . . G    
1025	. . . G    
1026	. . . G    
1027	. . . T    
1028	. . . T    
1029	. . . T    
1030	. . . G    
1031	. . . A    
1032	. . . T    
1033	. . . T    
1034	. . . C    
1035	. . . C    
1036	. . . T    
1037	. . . G    
1038	. . . C    
1039	. . . T    
1040	. . . T    
1041	. . . G    
1042	. . . G    
1043	. . . G    
1044	. . . C    
1045	. . . C    
1046	. . . A    
1047	. . . C    
1048	. . . A    
1049	. . . C    
1050	. . . A    
1051	. . . C    
1052	. . . A    
1053	. . . C    
1054	. . . T    
1055	. . . G    
1056	. . . A    
1057	. . . A    
1058	. . . T    
1059	. . . A    
1060	. . . T    
1061	. . . A    
1062	. . . T    
1063	. . . G    
1064	. . . T    
1065	. . . T    
1066	. . . A    
1067	. . . T    
1068	. . . T    
1069	. . . A    
1070	. . . C    
1071	. . . A    
1072	. . . A    
1073	. . . A    
1074	. . . C    
1075	. . . T    
1076	. . . A    
1077	. . . G    
1078	. . . T    
1079	. . . A    
1080	. . . C    
1081	. . . A    
1082	. . . C    
1083	. . . T    
1084	. . . C    
1085	. . . A    
1086	. . . A    
1087	. . . T    
1088	. . . C    
1089	. . . A    
1090	. . . T    
1091	. . . T    
1092	. . . T    
1093	. . . T    
1094	. . . A    
1095	. . . C    
1096	. . . A    
1097	. . . T    
1098	. . . G    
1099	. . . C    
1100	. . . A    
1101	. . . G    
1102	. . . T    
1103	. . . T    
1104	. . . G    
1105	. . . T    
1106	. . . G    
1107	. . . G    
1108	. . . T    
1109	. . . T    
1110	. . . T    
1111	. . . A    
1112	. . . C    
1113	. . . C    
1114	. . . A    
1115	. . . C    
1116	. . . C    
1117	. . . A    
1118	. . . C    
1119	. . . C    
1120	. . . A    
1121	. . . G    
1122	. . . G    
1123	. . . G    
1124	. . . G    
1125	. . . A    
1126	. . . A    
1127	. . . G    
1128	. . . C    
1129	. . . C    
1130	. . . T    
1131	. . . G    
1132	. . . T    
1133	. . . G    
1134	. . . G    
1135	. . . A    
1136	. . . A    
1137	. . . C    
1138	. . . A    
1139	. . . T    
1140	. . . G    
1141	. . . C    
1142	. . . A    
1143	. . . G    
1144	. . . T    
1145	. . . A    
1146	. . . A    
1147	. . . T    
1148	. . . A    
1149	. . . T    
1150	. . . G    
1151	. . . A    
1152	. . . C    
1153	. . . A    
1154	. . . G    
1155	. . . C    
1156	. . . G    
1157	. . . C    
1158	. . . T    
1159	. . . G    
1160	. . . C    
1161	. . . A    
1162	. . . G    
1163	. . . T    
1164	. . . G    
1165	. . . G    
1166	. . . G    
1167	. . . A    
1168	. . . G    
1169	. . . A    
1170	. . . A    
1171	. . . G    
1172	. . . A    
1173	. . . A    
1174	. . . T    
1175	. . . G    
1176	. . . G    
1177	. . . C    
1178	. . . C    
1179	. . . A    
1180	. . . G    
1181	. . . G    
1182	. . . C    
1183	. . . C    
1184	. . . G    
1185	. . . T    
1186	. . . G    
1187	. . . T    
1188	. . . T    
1189	. . . T    
1190	. . . T    
1191	. . . A    
1192	. . . G    
1193	. . . G    
1194	. . . T    
1195	. . . T    
1196	. . . T    
1197	. . . C    
1198	. . . A    
1199	. . . C    
1200	. . . T    
1201	. . . T    
1202	. . . C    
1203	. . . A    
1204	. . . C    
1205	. . . A    
1206	. . . G    
1207	. . . C    
1208	. . . C    
1209	. . . A    
1210	. . . T    
1211	. . . G    
1212	. . . T    
1213	. . . G    
1214	. . . C    
1215	. . . C    
1216	. . . T    
1217	. . . G    
1218	. . . G    
1219	. . . G    
1220	. . . T    
1221	. . . A    
1222	. . . G    
1223	. . . T    
1224	. . . G    
1225	. . . G    
1226	. . . G    
1227	. . . A    
1228	. . . A    
1229	. . . G    
1230	. . . G    
1231	. . . G    
1232	. . . G    
1233	. . . A    
1234	. . . G    
1235	. . . G    
1236	. . . T    
1237	. . . C    
1238	. . . C    
1239	. . . C    
1240	. . . A    
1241	. . . G    
1242	. . . C    
1243	. . . A    
1244	. . . A    
1245	. . . T    
1246	. . . C    
1247	. . . A    
1248	. . . T    
1249	. . . G    
1250	. . . T    
1251	. . . C    
1252	. . . C    
1253	. . . T    
1254	. . . C    
1255	. . . A    
1256	. . . A    
1257	. . . T    
1258	. . . G    
1259	. . . T    
1260	. . . G    
1261	. . . C    
1262	. . . T    
1263	. . . C    
1264	. . . C    
1265	. . . T    
1266	. . . T    
1267	. . . T    
1268	. . . A    
1269	. . . T    
1270	. . . G    
1271	. . . G    
1272	. . . C    
1273	. . . G    
1274	. . . C    
1275	. . . T    
1276	. . . T    
1277	. . . A    
1278	. . . G    
1279	. . . T    
1280	. . . T    
1281	. . . G    
1282	. . . T    
1283	. . . T    
1284	. . . A    
1285	. . . G    
1286	. . . T    
1287	. . . G    
1288	. . . C    
1289	. . . A    
1290	. . . T    
1291	. . . G    
1292	. . . T    
1293	. . . G    
1294	. . . C    
1295	. . . A    
1296	. . . C    
1297	. . . A    
1298	. . . C    
1299	. . . A    
1300	. . . C    
1301	. . . A    
1302	. . . C    
1303	. . . A    
1304	. . . C    
1305	. . . A    
1306	. . . C    
1307	. . . A    
1308	. . . C    
1309	. . . A    
1310	. . . C    
1311	. . . A    
1312	. . . C    
1313	. . . A    
1314	. . . C    
1315	. . . A    
1316	. . . C    
1317	. . . A    
1318	. . . C    
1319	. . . A    
1320	. . . C    
1321	. . . A    
1322	. . . C    
1323	. . . A    
1324	. . . C    
1325	. . . A    
1326	. . . C    
1327	. . . A    
1328	. . . C    
1329	. . . A    
1330	. . . C    
1331	. . . A    
1332	. . . C    
1333	. . . A    
1334	. . . C    
1335	. . . T    
1336	. . . G    
1337	. . . A    
1338	. . . T    
1339	. . . C    
1340	. . . C    
1341	. . . C    
1342	. . . A    
1343	. . . T    
1344	. . . G    
1345	. . . C    
1346	. . . T    
1347	. . . G    
1348	. . . A    
1349	. . . C    
1350	. . . G    
1351	. . . T    
1352	. . . G    
1353	. . . C    
1354	. . . C    
1355	. . . A    
1356	. . . A    
1357	. . . C    
1358	. . . A    
1359	. . . C    
1360	. . . C    
1361	. . . T    
1362	. . . A    
1363	. . . C    
1364	. . . C    
1365	. . . A    
1366	. . . A    
1367	. . . T    
1368	. . . C    
1369	. . . A    
1370	. . . C    
1371	. . . A    
1372	. . . C    
1373	. . . A    
1374	. . . G    
1375	. . . C    
1376	. . . C    
1377	. . . A    
1378	. . . G    
1379	. . . A    
1380	. . . G    
1381	. . . A    
1382	. . . G
```
